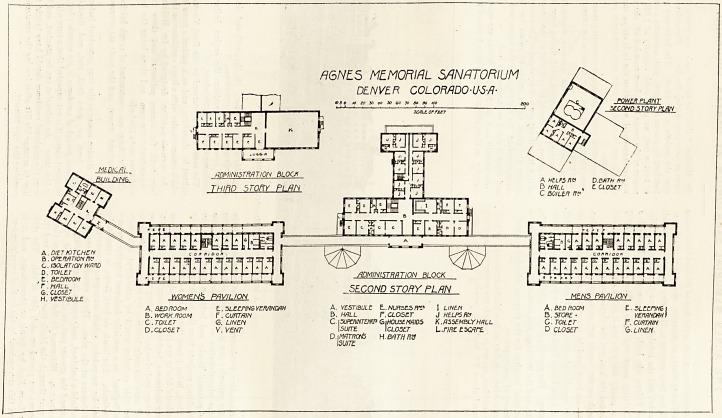# The Agnes Memorial Sanatorium at Denver, Colorado

**Published:** 1906-04-28

**Authors:** 


### THE AGNES MEMORIAL SANATORIUM AT DENVER, COLORADO.

THIS sanatorium, which is the first of its kind built in the Western United States of America, was opened in the year 1904. It has been erected in Montclair, which is a suburb of Colorado; and the inhabitants owe its existence to Mr. Lawrence Phipps who built it as a memorial to his mother whose Christian name was Agnes. The cost was 300,000 dollars, or nearly £60,000; but it is not stated whether this sum included the land and the furnishing or not.

It is built on dry, sandy soil, at an altitude of 5,400 feet above the sea-level, and in consequence of this height it commands magnificent views of the Rocky Mountain Range extending from Pike's Peak on the one hand to Long's Peak on the other. The grounds are beautifully laid out in lawns and walks, and the buildings and surroundings should form an almost ideal hospital for the purpose intended—namely, the treatment of consumptives.

The sanatorium consists of five blocks connected to each other by corridors, except as regards the power house, which has a tunnel about 500 feet long running between it and the administrative department. Of the remaining blocks two are for patients; one is the medical building and the other is the administrative which is placed almost in line with the patients' blocks and between them, while the medical building projects from the north-west corner of the women's block, being joined thereto by a corridor.

AGNES MEMORIAL SANATORIUM, DENVER, COLORADO.

The patients' blocks are two stories high, and each floor has twenty bedrooms, besides workroom, linen-room, bath-room, lavatory and closets. Regarding the latter they are not cut off from the main building by cross-ventilated passages, and this omission would be considered extremely faulty in England, in a specially designed hospital for any class of patients. The block, on both floors, is completely surrounded by verandahs, which arrangement constitutes the chief feature of the plan. These verandahs are wide enough to hold a bedstead, and, except in bad weather, the patients spend the greater part, or almost the whole, of the 24 hours thereon. Reference to the plan will show that a corridor runs through the block from end to end, and that the bedrooms are placed on each side of this. The main elevation faces East by South or thereabouts, and it is claimed for this arrangement by the medical superintendent, Dr. Holden, "that the direct sun's rays penetrate into each room at some time during the day." The staircase is placed near the middle of the block. The bedrooms are fourteen feet long by eleven feet wide, and, assuming a ceiling height of twelve feet, we get a cubic capacity of 1,850 feet. The angles of the rooms have been rounded off, the walls are of washable material, and when the beds are on the verandahs curtains can be used to render each bed-space separate from the others.

The medical building contains consulting-room, reception-room, chest, throat, and nose examination room, laboratory, $x$-ray room, etc.

The administrative block contains dining-hall, board-room, serving-room, kitchen, staff dining-room, and all the usual offices. It should be said that all the component parts of this department are well arranged. The second and third stories are given up to suites of rooms for the medical superintendent, the matron, and the nurses and domestics; and there is also a large assembly hall.

As already said the patients' blocks are connected by a corridor, and the administrative block is placed to the north-west side of the corridor. On the south-east side there are two large semicircular sun-rooms.

The power house contains the engine, boiler, laundry, sterilising-room, and crematory.

The sanatorium is warmed on the Paul Vacuum System, lighted by electricity, and each patient's room has a shaft which communicates with a large ventilator on the roof.

An open-air pavilion, designed by Dr. Holden, has also been built for women patients; and there are modern "house-tents" for men; and these bring the total accommodation up to 150 beds.

The buildings are constructed of rough brickwork faced with Portland cement, and the roofs are covered with red tiles.

Dr. Holden names as the four important factors of treatment: fresh air, good food, rest, and discipline. The minimum period of residence is six months; but, except in incipient cases, it is not expected that a cure will be effected in this time. An extension can be granted, and in any case even the minimum residence should have taught the patient how to arrange his life after leaving the sanatorium, and so complete the cure himself.

AGNES MEMORIAL SANATORIUM

DENVER COLORADO·U·S·A·

## Figures and Tables

**Figure f1:**
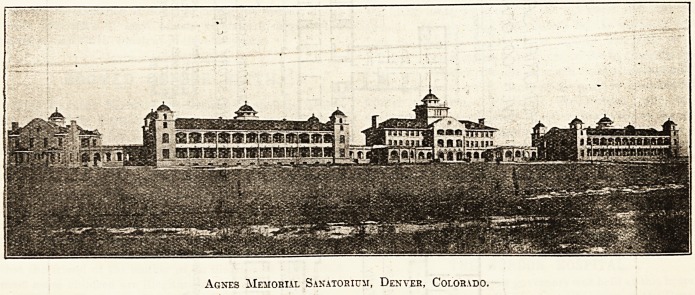


**Figure f2:**